# Psychophysiological responses to manual lifting of unknown loads

**DOI:** 10.1371/journal.pone.0247442

**Published:** 2021-02-26

**Authors:** Tamer M. Khalaf, Mohamed Z. Ramadan, Adham E. Ragab, Mohammed H. Alhaag, Khalil A. AlSharabi

**Affiliations:** 1 Department of Industrial Engineering, College of Engineering, King Saud University, Riyadh, Saudi Arabia; 2 Department of Mechanical Engineering, College of Engineering, Al-Azhar University, Cairo, Egypt; 3 Department of Electrical Engineering, College of Engineering, King Saud University, Riyadh, Saudi Arabia; Jonkoping University, SWEDEN

## Abstract

**Background:**

The handling of unknown weights, which is common in daily routines either at work or during leisure time, is suspected to be highly associated with the incidence of low back pain (LBP).

**Objectives:**

To investigate the effects of knowledge and magnitude of a load (to be lifted) on brain responses, autonomic nervous activity, and trapezius and erector spinae muscle activity.

**Methods:**

A randomized, within-subjects experiment involving manual lifting was conducted, wherein 10 participants lifted three different weights (1.1, 5, and 15 kg) under two conditions: either having or not having prior knowledge of the weight to be lifted.

**Results:**

The results revealed that the lifting of unknown weights caused increased average heart rate and percentage of maximum voluntary contraction (%MVC) but decreased average inter-beat interval, very-low-frequency power, low-frequency power, and low-frequency/high-frequency ratio. Regardless of the weight magnitude, lifting of unknown weights was associated with smaller theta activities in the power spectrum density (PSD) of the central region, smaller alpha activities in the PSD of the frontal region, and smaller beta activities in the PSDs of both the frontal and central regions. Moreover, smaller alpha and beta activities in the PSD of the parietal region were associated only with lifting of unknown lightweights.

**Conclusions:**

Uncertainty regarding the weight to be lifted could be considered as a stress-adding variable that may increase the required physical demand to be sustained during manual lifting tasks. The findings of this study stress the importance of eliminating uncertainty associated with handling unknown weights, such as in the cases of handling patients and dispatching luggage. This can be achieved through preliminary self-sensing of the load to be lifted, or the cautious disclosure of the actual weight of manually lifted objects, for example, through clear labeling and/or a coding system.

## Introduction

Low back pain (LBP) is the most prevalent work-related musculoskeletal disorder and is the leading cause of disability globally [1–3]. LBP incurs high costs in terms of medical treatment, lost work time, and low productivity [2, 4]. The severe consequences of LBP have motivated researchers to investigate its association with various aspects of manual lifting such as load magnitude, and load knowledge [5–11]. One aspect that is suspected of being highly associated with the incidence of LBP is the handling of unknown weights, which is common in daily routines either at work or during leisure time [6, 7, 12]. Examples of such situations are patient handling, luggage dispatching, refuse collecting, and mail distributing. An association between unexpected loading and LBP has been perceived, suggesting an elevated incidence of LBP when lifting unknown weights during refuse collecting or luggage handling [6, 7, 12].

Previous studies have investigated trunk muscle responses and spinal loading when lifting unknown loads and concluded that lifting unknown loads increases the risks of low back injury and balance loss based on the relationship between lifting unknown loads and increased trunk mechanical load, alteration of kinematic responses, and increased trunk muscle activities [6–9, 12–18].

One emerging technique for investigating human performances during physical activities is measuring brain responses via electroencephalography (EEG) [19–21]. EEG brain signals are associated with various cognitive and physical states, are sensitive to fluctuations in vigilance, and are significantly correlated with mental workload [22]. Such techniques consider human physical, cognitive, and affective capabilities and limitations when controlling and designing physical tasks [20, 21, 23]. EEG signals are a combination of brain rhythms at different frequency levels that fall into five frequency bands: 1) delta waves (1–3 Hz), 2) theta waves (4–7 Hz), 3) alpha waves (8–12 Hz), 4) beta waves (13–30 Hz), and 5) gamma waves (31–50 Hz).

Researchers have been investigating the emergence of activity within each frequency level and the associated physical and/or mental activity with the purpose of diagnosing, monitoring and assessing illnesses and human performance [24, 25]. Generally, delta waves are associated with unconsciousness, deep sleep, or catalepsy; theta waves are associated with creativity, spontaneity, distractibility, inattention, daydreaming, depression, and anxiety; alpha waves are associated with physical and mental relaxation as well as awareness of one’s surroundings; beta waves are associated with focusing, analysis, conscious alertness, tension, and fear; and gamma waves are associated with problem solving, learning, and facing cognitive challenges [24, 25].

Previous studies have indicated that activation of the brain in the frontal lobe can be used as a cognitive workload indicator [26]. The fluctuations in the power of both theta and alpha bands are associated with the performance of memory and complex cognitive [27, 28]. Decreased alpha band activities are associated with higher cognitive demands [29, 30]. Additionally, increased beta band power in both frontal and central lobes is associated with increased mental effort [31].

For the *θ*, *α*, *β*, and *γ* bands in the frontal, central, and parietal areas, the EEG power spectrum density (PSD) during medium load lifting tasks were found to be significantly greater than during low load lifting tasks, implying an increase in attention [32]. Cognitive processes such as self-monitoring were found to be associated with increased workload in the form of increased power in the waves in the 4–13 Hz range, which are associated with alert functioning [33].

Another emerging technique for investigating human performance during physical activities is the monitoring of heart rate variability (HRV), which represents the periodic variations in the heart rate (HR), and is an indicator of the activity level of the cardiovascular autonomic function [34]. HRV depends on age, body mass index, sex, diet, and physical and mental activity [35, 36]. The HRV indices used for analysis come from both the time and frequency domains. The indices from the time domain are the average inter-beat (RR) interval (mRR), average heart rate (mHR), standard deviation of normal-to-normal RR intervals (SDNN), root mean square of successive differences in RR intervals (RMSSD), number of pairs of successive RR intervals that differ by more than 50 ms (NN50), and proportion of NN50 divided by the total number of RR intervals (pNN50). The indices from the frequency domain are the PSD in the very-low-frequency band (VLF = 0–0.04 Hz), low-frequency band (LF = 0.04–0.15 Hz), and high-frequency band (HF = 0.15–0.4 Hz), and the total power (TP = 0–0.4 Hz). The HF power have been associated with vagal activity [37], and the LF/HF ratio is an indicator of sympathetic–parasympathetic balance [38]. Theoretically, the HF component is more sensitive to physical effort and is lower during physical tasks, the LF component is sensitive to overall strain (either physical or mental, but is typically higher for mental stress), and the LF/HF ratio indicates autonomic balance (for any kind of activity) [36, 38–40].

To the best of our knowledge, the lifting of unknown loads based on brain’s EEG or HRV responses during regular daily activities has not been investigated. Investigating the EEG responses to lifting unknown loads should reveal the contributions of the brain during such daily activities, rather than simply focusing on peripheral outcomes such as forces and muscle activity. Investigating HRV responses during the lifting of unknown loads should also elucidate cardiovascular autonomic functions during such tasks. The objective of this study was to investigate the effects of knowledge and magnitude of a load (to be lifted) on brain responses, autonomic nervous activity, and trapezius and erector spinae muscle activity.

## Materials and methods

The study was approved by the Human Participants Review Sub-committee of the Institutional Review Board of King Saud University, College of Medicine, and King Khalid University Hospital (ID: E-19-4467).

### Experimental design

A repeated-measures design, with two independent variables and three response variables was used in this study. The independent variables were weight knowledge with two levels (known weight and unknown weight) and weight magnitude with three levels (1.1 kg, 5 kg, and 15 kg), resulting in six experimental conditions corresponding to the six combinations of the independent variables’ levels, which were assigned in a completely randomized order. The dependent variables were the PSDs of the four EEG bands (*α*, *β*, *θ*, and *γ*), HRV, and muscle activity as a percent of maximum voluntary contraction (%MVC) for the trapezius and erector spinae muscles.

### Participants

Ten healthy male university students with a mean age (standard deviation) of 30.5 (1.21) years, a mean height of 168.7 (2.64) cm, and a mean weight of 75.18 (8.37) kg voluntarily participated in this study. None of the participants had experience in manual lifting tasks, and none had a history of neurological disorders, back pain, or any other musculoskeletal injury. All participants signed a consent form approved by the Human Participants Review Sub-committee of the Institutional Review Board of King Saud University, College of Medicine, and King Khalid University Hospital (ID: E-19-4467).

### Equipment

Three two-handled boxes (39cm×27cm×19cm) were used for the lifting loads. An eight-channel Biomonitor ME6000, MT-ECG-1 preamplifier, four-channel EEG amplifier for the ME6000, and the Mega Win3.0.1 software (Mega Electronics Ltd., Kuopio, Finland) were used to record physiological signals (four channels to record EEG signals, two channels to record electrocardiography (ECG) signals, and two channels to record electromyography (EMG) signals). An EMOTIV EEG headset was used for holding the EEG electrodes in place. In addition, the Kubios HRV Software v2.2 (University of Western Finland, Finland) was used to compute the HRV.

### Physiological response measurements

#### EMG response measure

Standard procedures were followed for the placement of the Ag/AgCl solid adhesive pre-gelled electrodes on the right erector spinae and middle trapezius muscles of the participants. EMG signals were recorded at a sampling rate of 1000 Hz. Low-frequency artefacts were removed using a band-pass filter with a frequency range of 20–500 Hz. A 50 Hz notch filter was then used to remove the 50 Hz power line interference in the recorded EMG signals. Muscle activities during maximum voluntary contraction (MVC) for the two muscles investigated were recorded, to be used for normalizing the muscle activities recorded under the experimental conditions.

#### ECG response measure

Three ECG Ag/AgCl electrodes were placed on the participants’ chests to reduce the occurrence of muscle contraction artefacts during lifting. The ECG signals were recorded at a sampling rate of 1000 Hz. The Kubios HRV Software v2.2 was used for processing the ECG signals [41]. Then both time domain parameters and frequency domain parameters of the HRV were calculated [time domain parameters: mRR, mHR, standard deviation of all RR intervals (SDRR), standard deviation of the mRR intervals in all segments of the recordings (SDHR), RMSSD, NN50, and pNN50; frequency domain parameters: VLF, LF, HF, and LF/HF]. Power is calculated as time squared in milliseconds divided by frequency in Hertz (ms^2^/Hz).

#### EEG response measure

The international standards of the 10–20 for EEG electrode placement was followed [42]. EEG signals were recorded from the right side of the head at three positions: 1) F4: frontal cortex which is responsible for attention, judgment, and motor planning, 2) Fc6: central cortex which is responsible for the sensorimotor system, and 3) P8: parietal region, where cognitive processing occurs. The EEG signals were amplified and recorded at a sampling rate of 1000 Hz. The recorded EEG signals were visually monitored at all times for any suspicious artefacts. For the purpose of artefact removal, EEG signals associated with specific predetermined body movements (explained in the Experimental Procedures sub-section) was separately recorded to be used later in the artefact subspace reconstruction method implemented to remove nonstationary high-variance signals from the recorded raw EEG signals during performing the experimental tasks and to rebuild any missing data using a spatial mixing matrix, assuming volume conduction [43]. Next, a low-pass four-pole elliptic filter with a cut-off frequency of 50 Hz was used to remove the power line noise and any other high-frequency noise. The filtered EEG signals were then separated into their individual bands (*θ*, *α*, *β*, and *γ*) using a multilevel discrete wavelet transform. A digital FFT-based power spectrum analysis (Welch technique, Hanning windowing function, no phase shift) was used to compute the PSD of EEG rhythms with a 1 Hz frequency resolution, ranging from 0.5 to 50 Hz. The calculated PSDs of the four bands *θ*, *α*, *β*, and *γ* are the response variables in the three selected regions.

### Experimental procedures

Each participant received a clear description of the objectives of the study and then signed a consent form. The participants’ demographic data, age, and anthropometric dimensions were collected. They were then equipped with the ECG, EEG, and EMG systems and prepared for the experiment. Then muscle activities at the MVC for both the erector spinae muscle and the middle trapezius muscle were recorded. A five-minute EEG signal containing artefacts was next recorded. Artefacts include eye blinking, chewing, and body motion such as arm, leg, and head movements, and physical tasks such as lifting (1-minute eye closed, one-minute eye blinking, one-minute chewing, one-minute face and arm movements, and one minute performing lifting task). These signals were used during data analysis to remove such artefacts from the EEG signals.

Participants were instructed on how to perform squat lifting. The lifting experiment started with each participant squat lifting a box from the floor to a height of 70 cm in the sagittal plan. The perpendicular distance between the line connecting the medial malleoli and the projected center of mass of the lifted box was maintained constant at 45 cm across all experimental conditions. Three identical boxes, with the three different weights of interest, were used to eliminate the possibility that the participants could anticipate the box weight. The box was changed randomly six times for the six lifts. In three of the lifts, the participants were told the box weight before lifting. A two-minute rest followed each trial to minimize fatigue.

### Statistical analysis

A Two-Way Repeated Measures ANOVA was performed to assess the main and interaction effects of the independent variables at a significance level of *α* ≤ 0.05. Post-hoc tests were used to further analyze significant main effects of the weight magnitude factor with Bonferroni adjustments for multiple comparisons. For significant interaction effects, paired *t*-test comparisons were performed to independently assess the effects of weight knowledge at each weight magnitude level and weight magnitude at each weight knowledge level. Both Shapiro Wilk and Kolmogorov-Smirnova tests of normality were performed for all the response data under all the experimental conditions to assure that the normality assumption is satisfied. In addition, Mauchly’s test of Sphericity was performed for all the response data under all the experimental conditions to assure that the homogeneity of variance assumption is satisfied. Whenever the homogeneity of variance assumption was not satisfied, Greenhouse-Geisser correction to the degrees of freedom was considered to adjust for the lack of sphericity. The statistical analysis was performed using SPSS Version 23.

## Results

Both Shapiro Wilk and Kolmogorov-Smirnova tests of normality were not significant for all the response data under all the experimental conditions implying that the normality assumption is satisfied. In addition, Mauchly’s test of Sphericity was not significant for all the reported significant effects implying that the homogeneity of variance assumption is satisfied except for the weight magnitude main effects for the %MVC of the erector spinae and the Greenhouse-Geisser correction to the degrees of freedom was considered as reported in the following results.

### EMG response measure

For the trapezius muscle, the interaction between weight knowledge and weight magnitude had a significant effect on the %MVC [F(2,18) = 5.1, *p*<0.018, *ƞ*^*2*^ = 0.36]. A simple effects analysis revealed that at both the 1.1 kg and the 15 kg weights, the %MVC of the trapezius muscle was significantly higher for the unknown weights than for the known weights [*p*<0.001 and *p*<0.005, respectively]. For the known condition, the %MVC of the trapezius muscle was significantly lower for the 1.1 kg than for both the 5 kg and the 15 kg weights, [*p*<0.0001 and *p*<0.0001, respectively], and for the 5 kg than for the 15 kg weights [*p*<0.0001]. For the unknown condition, the %MVC of the trapezius muscle was significantly higher for the 15 kg weight than for both the 1.1 kg and the 5 kg weights, [*p*<0.003 and *p*<0.003, respectively]. The means, standard deviations (SD), *p*-values, and effect sizes (*ƞ*^2^) of the %MVC of both the trapezius and the erector spinae muscles are summarized in Table 1.

**Table 1:** Means, Standard Deviations (SD), *p*-values, and effect sizes (*ƞ*^2^) of the %MVC of both the Trapezius and the Erector Spinae muscles.

Parameters	Mean (SD)						Statistics *p*-value (*ƞ*^2^)		
Weight Knowledge	Known			Unknown			Weight knowledge	Weight magnitude	Interaction
Weight Magnitude	1.1 kg	5 kg	15 kg	1.1 kg	5 kg	15 kg			
**Trapezius muscle**	4.2 (2.7)	14.6 (7.3)	20.9 (8.9)	12.2 (6.8)	16.0 (9.2)	29.6 (13.3)	**0.001 (0.71)** *	**0.0001 (0.74)** *	0.018 (0.36)*
**Erector Spinae muscle**	40.4 (18.5)	48.9 (22.1)	73.7 (37.9)	47.7 (27.1)	59.2 (26.9)	85.4 (43.7)	**0.035 (0.41)** *	**0.001 (0.68)** **	0.38 (0.10)

* Significance level at p < 0.05.

** Greenhouse-Geisser correction used because Muchly’s test was significant for this variable.

**Fig 1:**
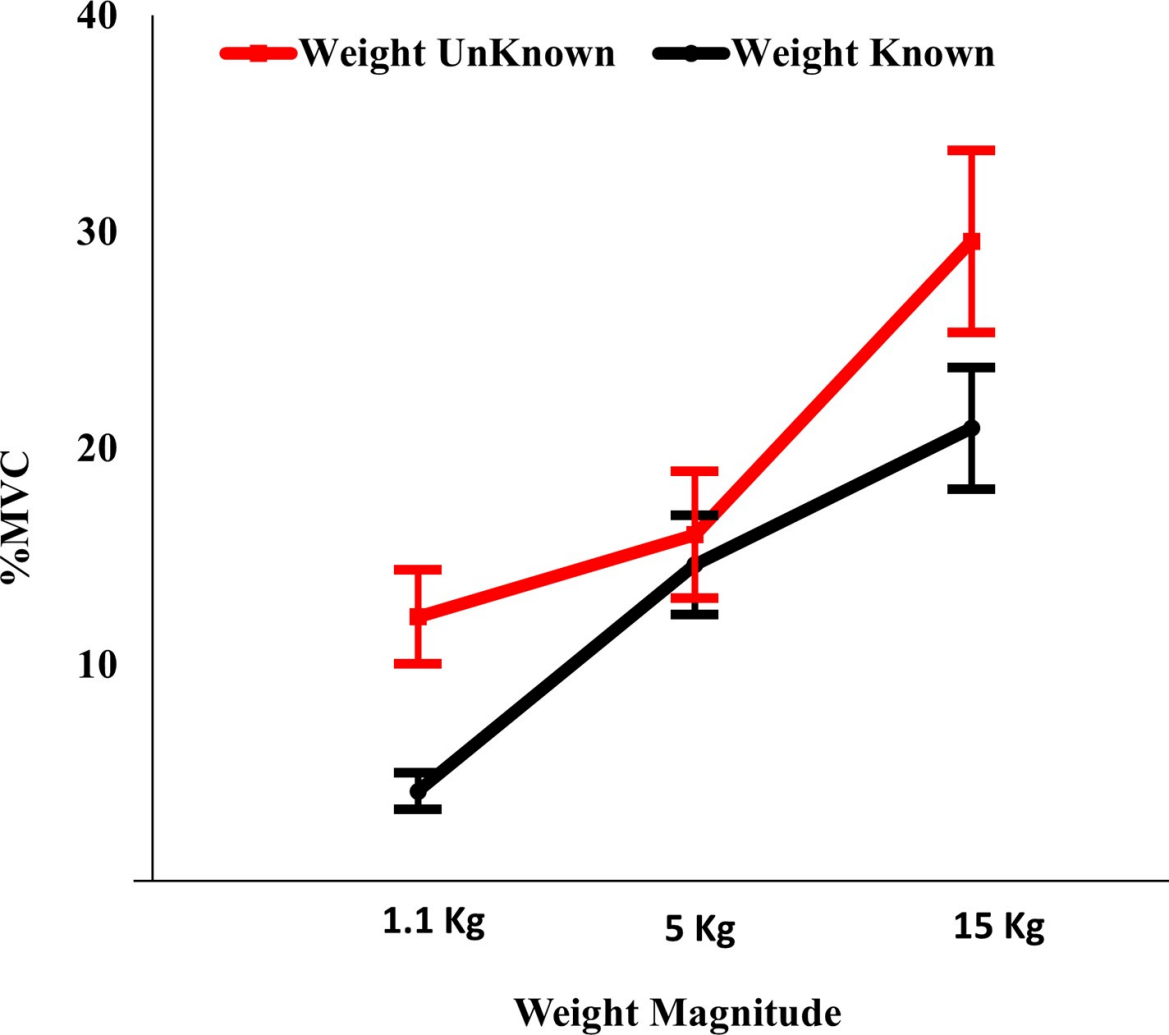
Effects of weight knowledge and weight magnitude on %MVC of trapezius muscle.

The means and standard errors of the %MVC of the trapezius muscle are represented in Fig 1.

Weight magnitude had a significant effect on the %MVC of the erector spinae muscle [F(1.23,11.68) = 18.9, *p*<0.001, *ƞ*^*2*^ = 0.68]. The post-hoc analysis revealed that the %MVC of the erector spinae muscle was significantly higher for 1) the 15 kg weight compared to both the 1.1 kg and the 5 kg weights [*p*<0.001 and *p*<0.004, respectively] and 2) the 5 kg weight compared to the 1.1 kg weight [*p*<0.011]. Weight knowledge also had a significant effect on the %MVC of the erector spinae muscle [F (1,9) = 6.2, *p*<0.035, *ƞ*^2^ = 0.41], where lifting unknown weights was associated with a higher %MVC.

### ECG responses

For the HRV indices in the time domain, weight knowledge had a significant effect on both the mRR [F(1,9) = 10.86, *p*<0.009, *ƞ*^*2*^ = 0.55] and the mHR [F(1,9) = 8.5, *p*<0.017, *ƞ*^*2*^ = 0.49]. Lower mRR values and higher mHR values were associated with lifting unknown weights. Weight magnitude had a significant effect on the mHR [F(2,18) = 4.35, *p*<0.029, *ƞ*^*2*^ = 0.33]. The post-hoc analysis revealed that the mHR was significantly lower for the 1.1 kg weight than for the 15 kg weight [*p*<0.044]. Weight magnitude had no significant effect on the mRR. Weight knowledge, weight magnitude, and their interactions also had no significant effect on the SDRR, SDHR, RMSSD, NN50, or pNN50.

**Fig 2:**
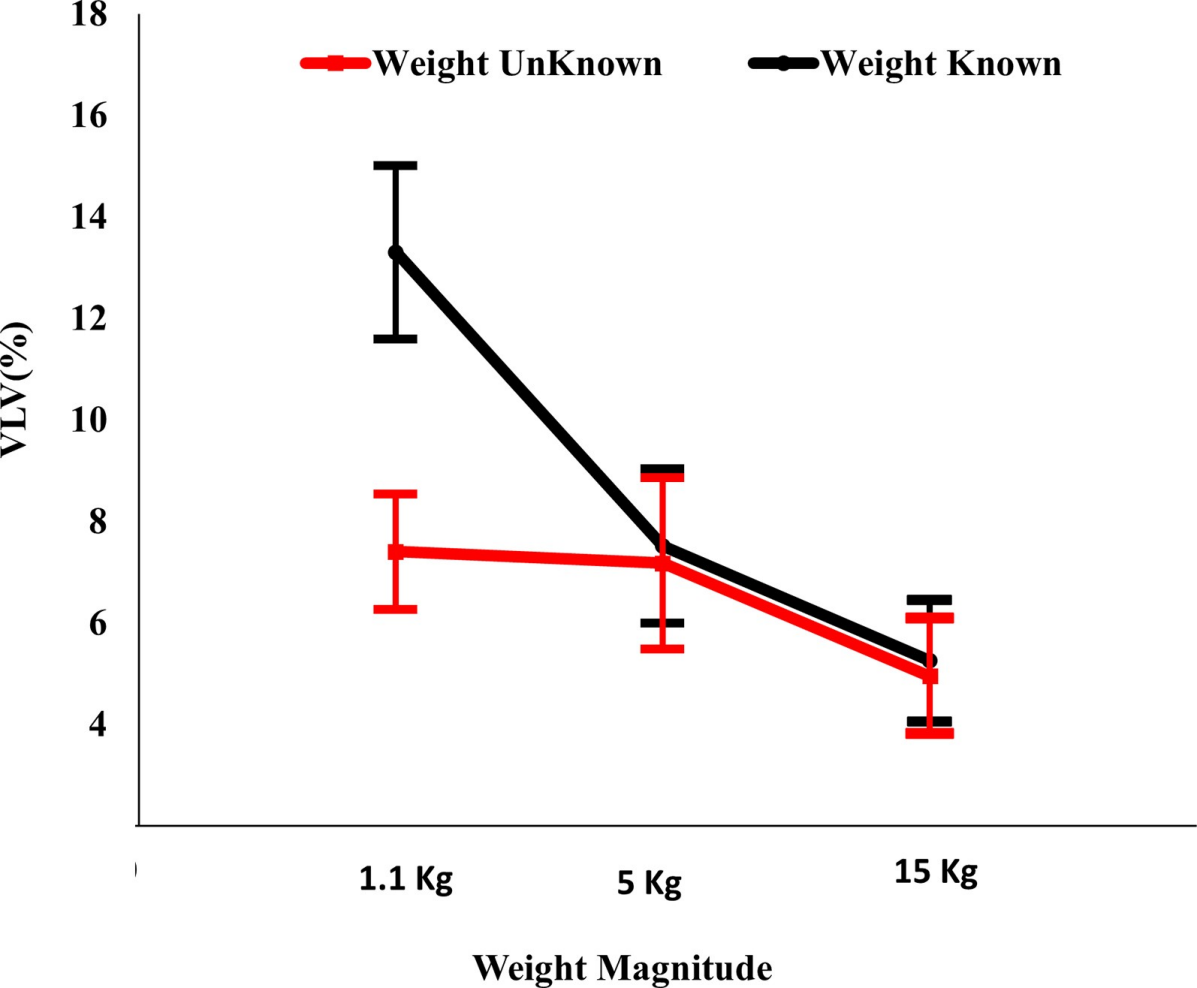
Effects of weight knowledge and weight magnitude on VLF power.

For the HRV indices in the frequency domain, the interaction between weight knowledge and weight magnitude had a significant effect on VLF power [F(2,18) = 4.17, *p*<0.033, *ƞ*^*2*^ = 0.32]. The simple effects analysis revealed that at the 1.1 kg weight condition, the VLF power was significantly higher for the known weight than for the unknown weight [*p*<0.004]. Under the known weight condition, the VLF power was also significantly higher for the 1.1 kg weight than for both the 5 kg and the 15 kg weights [*p*<0.029 and *p*<0.002, respectively]. The means and standard errors of the VLF power are represented in Fig 2.

Weight knowledge had a significant effect on both the LF power [F(1,9) = 7.31, *p*<0.024, *ƞ*^*2*^ = 0.45] and the LF/HF ratio [F (1,9) = 8.94, *p*<0.015, *ƞ*^*2*^ = 0.5]. Both the LF power, and the LF/HF ratio were significantly lower when lifting unknown weights than when lifting known weights. These results revealed that lifting unknown weights decreases VLF power, LF power, and LF/HF ratio compared to lifting known weights.

Weight magnitude had a significant effect on the LF power [F(2,18) = 5.85, *p*<0.011, *ƞ*^*2*^ = 0.39]. The post-hoc analysis revealed that the LF power was significantly higher for the 1.1 kg weight than for the 15 kg weight [*p*<0.032]. Weight magnitude also had a significant effect on the LF/HF ratio [F(2,18) = 9.13, *p*<0.002, *ƞ*^*2*^ = 0.5]. The post-hoc analysis revealed that the LF/HF ratio was significantly higher for the 1.1 kg weight than for the 15 kg weight [*p*<0.004]. Weight knowledge, weight magnitude, and their interaction had no significant effect on the HF power. The means, standard deviations (SD), *p*-values, and effect sizes (*ƞ*^2^) of the heart rate variability (HRV) indices are summarized in Table 2.

**Table 2:** Means, Standard Deviations (SD), p-values, and effect sizes (*ƞ*^2^) of the Heart Rate Variability (HRV) indices.

Parameters	Mean (SD)						Statistics *p*-value (*ƞ*^2^)		
Weight Knowledge	Known			Unknown			Weight Knowledge	Weight Magnitude	Interaction
Weight Magnitude	1.1 kg	5 kg	15 kg	1.1 kg	5 kg	15 kg			
**mRR (ms)**	691.5 (75.6)	688.4 (78.0)	666.0 (67.2)	671.6 (85.2)	664.1 (80.3)	652.7 (52.6)	0.009 (0.55)*	0.025 (0.34)*	0.804 (0.24)
**SDRR (ms)**	40.7 (17.4)	44.5 (15.3)	43.2 (19.8)	35.0 (12.4)	35.9 (19.6)	37.7 (16.9)	0.15 (0.21)	0.754 (0.03)	0.958 (0.01)
**mHR (bpm)**	88.0 (8.9)	90.0 (9.3)	91.4 (9.2)	90.9 (10.7)	92.3 (8.3)	93.4 (8.1)	0.017 (0.49)*	0.029 (0.33)*	0.93 (0.01)
**SDHR (ms)**	4.6 (0.9)	5.9 (2.4)	5.94 (3.2)	5.2 (2.4)	4.4 (2.3)	4.8 (2.1)	0.30 (0.12)	0.67 (0.04)	0.41 (0.1)
**RMSSD (ms)**	49.4 (19.5)	48.4 (26.0)	52.3 (31.1)	41.6 (18.5)	42.2 (24.0)	44.5 (22.1)	0.297 (0.12)	0.821 (0.02)	0.99 (0.01)
**NN50**	2.7 (1.6)	2.6 (2.4)	2.1 (1.5)	2.3 (1.4)	2.2 (1.9)	2.5 (1.6)	0.721 (0.02)	0.939 (0.01)	0.7 (0.04)
**pNN50 (%)**	23.0 (14.2)	21.6 (21.6)	15.2 (10.5)	18.3 (11.4)	16.6 (13.5)	19.6 (12.7)	0.55 (0.04)	0.825 (0.02)	0.51 (0.07)
**VLF (ms**^**2**^**/Hz)**	13.3 (5.4)	7.5 (4.8)	5.3 (3.8)	7.4 (3.6)	7.2 (5.3)	5.0 (3.6)	0.020 (0.47)*	0.005 (0.44)*	0.033 (0.32)*
**LF (ms**^**2**^**/Hz)**	43.7 (11.3)	33.2 (16.3)	27.0 (12.4)	30.3 (12.1)	27.7 (10.3)	22.7 (14.9)	0.024 (0.45)*	0.011 (0.4)*	0.484 (0.08)
**HF (ms**^**2**^**/Hz)**	42.8 (15.4)	59.3 (20.6)	70.1 (17.6)	59.5 (18.8)	58.7 (19.0)	59.7 (23.9)	0.601 (0.032)	0.087 (0.24)	0.107 (.22)
**LF/HF**	1.2 (0.6)	0.7 (0.5)	0.4 (0.3)	0.6 (0.4)	0.5 (0.3)	0.4 (0.3)	0.015 (0.5)*	0.002 (0.50)*	0.214 (0.16)

* Significance level at *p* < 0.05.

### EEG response

**Fig 3:**
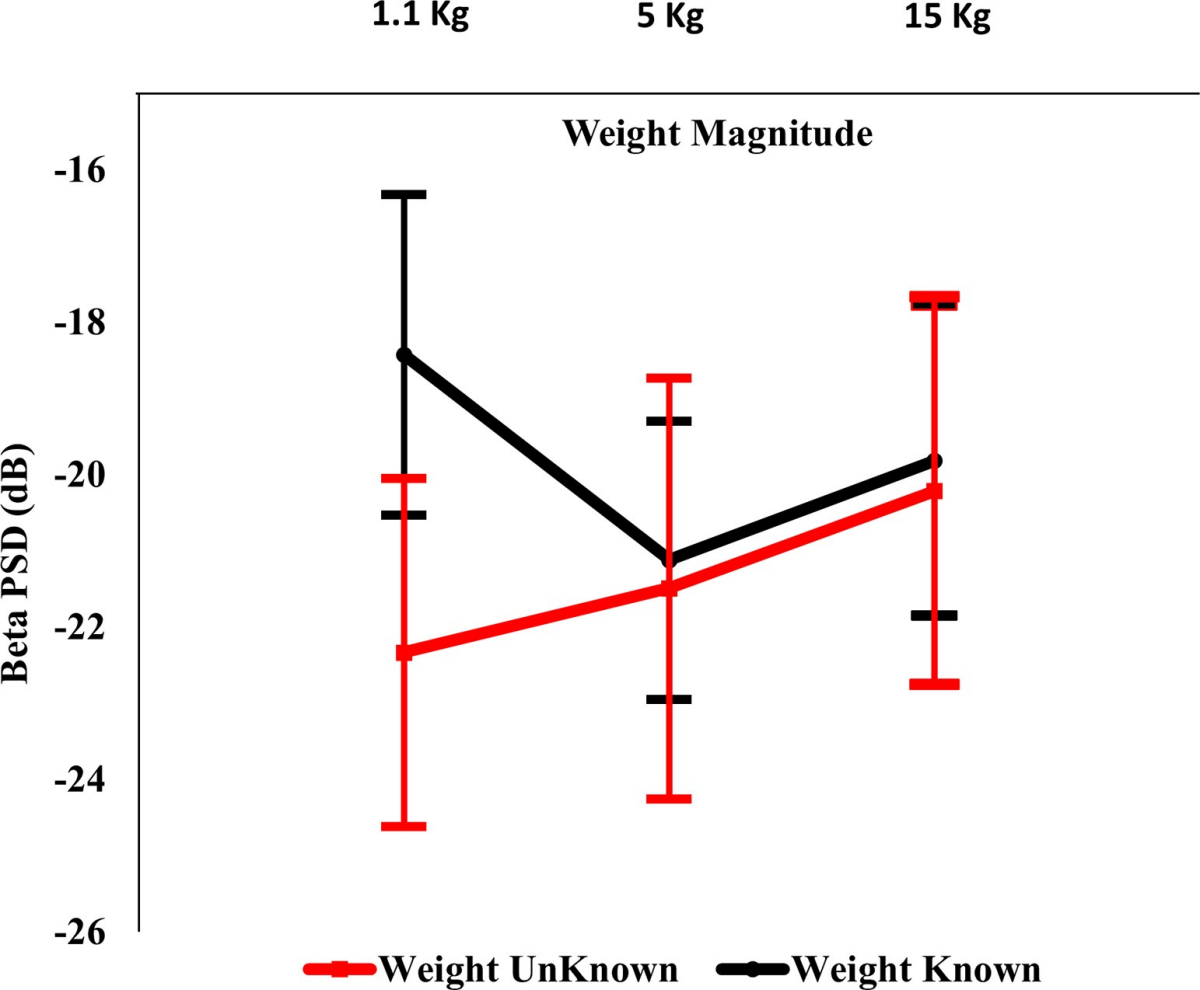
Effects of weight knowledge and weight magnitude on parietal region β band PSD.

The interaction between weight knowledge and weight magnitude had a significant effect on the *β* band PSD of the parietal region of the brain [F(2,18) = 4.46, *p*<0.027, *ƞ*^*2*^ = 0.33]. The simple effects analysis revealed that at the 1.1 kg weight condition, the *β* band PSD of the parietal region was significantly lower for the unknown weight than for the known weight [*p*<0.014]. Under the unknown condition, the *β* band PSD of the parietal region was also significantly lower for the 1.1 kg weight than for the 15 kg weight [*p*<0.049]. Means and standard errors of the *β* band PSD of the parietal region are represented in Fig 3.

Weight knowledge had a significant effect on the *α* band PSD of the frontal region of the brain [F(1,9) = 5.55, *p*<0.043, *ƞ*^*2*^ = 0.38], where lifting unknown weights was associated with a smaller *α* band PSD. In addition, weight knowledge had a significant effect on the *β* band PSD of both the frontal and central regions of the brain [frontal: F(1,9) = 9.64, *p*<0.013, *ƞ*^*2*^ = 0.52, and central: F(1,9) = 6.004, *p*<0.037, *ƞ*^*2*^ = 0.4], where lifting unknown weights was associated with a smaller *β* band PSD for both the frontal and central regions. In addition, weight knowledge had a significant effect on the *θ* band PSD of the central region of the brain [F(1,9) = 5.27, *p*<0.047, *ƞ*^*2*^ = 0.37], where lifting unknown weights was associated with a smaller *θ* band PSD.

Weight knowledge, weight magnitude, and their interactions had no significant effect on the *θ* band PSD of the frontal or parietal regions of the brain. Weight knowledge, weight magnitude, and their interactions had no significant effect on the *γ* band PSD of all brain regions. The means, standard deviations (SD), *p*-values, and effect sizes (*ƞ*^2^) of the power spectrum density (PSD) of the four EEG bands in the three brain regions are summarized in Table 3. All significant contrasts are summarized in Tables 4–6.

**Table 3:** Means, Standard Deviations (SD), *p*-values, and effect sizes (*ƞ*^2^) of the Power Spectrum Density (PSD) of the four EEG bands in the three brain regions.

Parameters		Mean (SD)						Statistic *p*-value (*ƞ*^2^)		
Weight Knowledge		Known			Unknown			Weight Knowledge	Weight Magnitude	Interaction
Weight Magnitude		1.1 kg	5 kg	15 kg	1.1 kg	5 kg	15 kg			
**Frontal**	**Theta**	-19.8 (9.5)	-20.3 (8.0)	-21.9 (9.8)	-23.3 (7.9)	-23.9 (6.7)	-20.8 (8.1)	0.123 (0.24)	0.956 (0.01)	0.185 (0.17)
	**Alpha**	-20.4 (9.4)	-21.9 (7.7)	-22.4 (8.9)	-23.9 (8.7)	-24.6 (6.8)	-25.0 (8.7)	0.043 (0.38)*	0.486 (0.08)	0.906 (0.01)
	**Beta**	-17.6 (8.9)	-19.6 (7.2)	-18.5 (8.1)	-21.7 (7.2)	-21.6 (7.3)	-22.5 (6.7)	0.013 (0.52)*	0.763 (0.03)	0.655 (0.05)
	**Gamma**	-20.6 (8.5)	-21.9 (6.9)	-20.4 (6.4)	-23.4 (7.0)	-23.1 (6.4)	-22.5 (7.2)	0.074(0.31)	0.672 (0.04)	0.777 (0.03)
**Central**	**Theta**	-21.7 (9.7)	-19.3 (8.9)	-22.1 (10.0)	-22.6 (7.6)	-24.1 (7.7)	-21.7 (8.8)	0.047 (0.37)*	0.939 (0.01)	0.213 (0.16)
	**Alpha**	-23.3 (10.6)	-23.5 (8.7)	-22.9 (9.8)	-23.9 (8.4)	-25.3 (8.2)	-23.1 (8.9)	0.302 (0.12)	0.354 (0.11)	0.677 (0.04)
	**Beta**	-19.6 (9.8)	-19.9 (8.2)	-19.2 (8.9)	-21.2 (8.9)	-21.8 (8.7)	-21.7 (9.4)	0.037 (0.40)*	0.923 (0.01)	0.974 (0.003)
	**Gamma**	-22.4 (9.8)	-21.6 (7.5)	-21.0 (7.1)	23.3 (7.2)	-23.2 (8.5)	-22.3 (8.5)	0.234 (015)	0.575 (0.06)	0.949 (0.01)
**Parietal**	**Theta**	-18.8 (8.2)	-21.1 (6.9)	-22.3 (8.5)	-22.8 (8.5)	-22.1 (8.7)	-21.2 (8.5)	0.240(0.15)	0.623 (0.05)	0.113 (0.22)
	**Alpha**	-20.1 (7.8)	-24.1 (7.3)	-23.8 (7.8)	-24.8 (8.1)	-24.0 (8.9)	-23.9 (8.0)	0.067 (0.33)	0.273 (0.13)	0.070 (0.31)
	**Beta**	-18.4 (6.6)	-21.1 (5.8)	-19.8 (6.6)	-22.3(7.2)	-21.5 (8.7)	20.2 (8.0)	0.108 (0.26)	0.33 (0.12)	0.027 (0.33)*
	**Gamma**	-20.4 (6.0)	-22.4 (4.7)	-21.2 (5.2)	-24.1 (6.6)	-22.3 (8.0)	-22.1 (7.4)	0.189 (0.18)	0.654 (0.05)	0.06 (0.27)

* Significance level at *p* < 0.05.

**Table 4:** Summary of significant contrasts where the interaction between the two independent variables was significant.

**% MVC Trapezius**	1.1 kg: Known < Unknown (*p* < 0.001)
	15 kg: Known < Unknown (*p* < 0.005)
	Known: 1.1 kg < 5 kg (*p* < 0.0001)
	Known: 1.1 kg < 15 kg (*p* < 0.0001)
	Known: 5 kg < 15 kg (*p* < 0.0001)
	Unknown: 1.1 kg < 15 kg (*p* < 0.003)
	Unknown: 5 kg < 15 kg (*p* < 0.003)
**VLF power**	1.1 kg: Known > Unknown (*p* < 0.004)
	Known: 1.1 kg > 5 kg (*p* < 0.029)
	Known: 1.1 kg > 15 kg (*p* < 0.002)
**Parietal *β* PSD**	1.1 kg: Known > Unknown (*p* < 0.014)
	Unknown: 1.1 kg < 15 kg (*p* < 0.049)

**Table 5:** Summary of significant contrasts where the main effects of the weight knowledge was significant.

**% MVC Erector Spinae**	Known < Unknown (*p* < 0.035)
**mHR**	Known < Unknown (*p* < 0.017)
**mRR**	Known > Unknown (*p* < 0.009)
**LF power**	Known > Unknown (*p* < 0.024)
**LF/HF ratio**	Known > Unknown (*p* < 0.015)
**Frontal *α* PSD**	Known > Unknown (*p* < 0.043)
**Frontal *β* PSD**	Known > Unknown (*p* < 0.013)
**Central *β* PSD**	Known > Unknown (*p* < 0.037)
**Central *θ* PSD**	Known > Unknown (*p* < 0.036)

**Table 6:** Summary of significant contrasts where the main effects of the weight magnitude was significant.

**% MVC Erector Spinae**	1.1 kg < 5 kg (*p* < 0.011)
	1.1 kg < 15 kg (*p* < 0.001)
	5 kg < 15 kg (*p* < 0.004)
**mHR**	1.1 kg < 15 kg (*p* < 0.044)
**LF power**	1.1 kg > 15 kg (*p* < 0.032)
**LF/HF ratio**	1.1 kg > 15 kg (*p* < 0.004)

## Discussions

This study investigated the brain EEG’s PSDs, cardiovascular autonomic functions and muscle activity responses associated with two factors of interest, which were weight magnitude with three levels representing light, medium, and heavy weights, and weight knowledge. In this study, for the erector spinae muscle, higher muscle activities were associated with lifting greater weight magnitudes and with lifting unknown weights, as expected and as indicated in the literature [2, 7, 9, 13, 14]. The interaction between both factors was not, however, influential. These results imply that the erector spinae muscle activities will increase with increased loading, regardless of whether these loads are known or not. Similarly, the erector spinae muscle activities will increase with uncertainty about the loads being lifted regardless of whether these loads are light, medium, or heavy.

For the trapezius muscle, the interaction between both factors of interest was significant where higher muscle activities were associated with lifting unknown weights under the light and heavy conditions of the weight magnitude factor but not the medium weight condition. This finding implies an added workload to the task due to an uncertainty about the magnitude of the load to be lifted when associated with lifting light loads or heavy loads but not medium loads. In industrial settings, although the lifted loads might be within certain acceptable value according to NIOSH lifting equation, we suspect that uncertainty might induce some sort of additional mental workload that, in conjunction with the presented physical workload due to the amount of load lifted, might push the task demands beyond the capabilities and probably exceeding the limitations of the workers.

This may suggest that with an uncertainty about the magnitude of the load, the trapezius muscle approaches the lifting task with an activation level that is pre-set to a medium load value. When the expectation matches the reality of the lifted load, knowledge makes no difference, but when the actually lifted weight is different (either higher or lower), the trapezius muscle response manifested itself in the form of higher activation, implying increased loading of the muscle. By investigating the differences among load levels under each knowledge condition separately, it was found that higher trapezius muscle activities were associated with higher load levels.

The results of HRV measures revealed that an increase in the mHR values and decrease in mRR values, VLF power, LF power, and LF/HF ratio are associated with lifting unknown and heavier weights. The increase in physical demand imposed by increasing the load magnitude and not knowing the weight to be lifted manifested itself in the form of significant changes in the above HRV parameters. These results introduce the HRV as a valuable tool for the monitoring, and assessment of human performance [34–37, 39].

The findings of this research suggest a decrease in the LF power as a result of increasing the physical demand, which is in agreement with the findings of Kamath et al. [39] and Perini and Veicsteinas [40], but is in contrast to the findings of Satya [36] and Tarvainen et al. [38]. Similarly, the LF/HF ratio decreased as a result of increasing the physical demand, which is in contrast to Kamath et al. [39], Satya [36], and Tarvainen et al. [38]. The contradiction in the results obtained can be justified by the fact that in the current research, the level of physical demand is considerably lower, being six lifts for the entire manual lifting session compared to the load and duration of the other studies. The manner in which participants responded systemically to lifting the unknown weights resulted in an increase in autonomic activities, especially in the cases of heavier weights.

In this study, the lifting of unknown weights was associated with a smaller *θ* band PSD of the central region, a smaller *α* band PSD of the frontal region, and a smaller *β* band PSD of both the frontal and the central regions, regardless of the weight magnitude. In addition, smaller *β* band PSD values for the parietal region was associated only with lifting unknown light weights. Lifting objects of unknown weight can increase the activation of the sensorimotor areas of the brain, particularly for weights of lower magnitudes, as a result of central adaptation counteraction. These findings are consistent with those in the literature regarding decreased PSD values for various EEG bands (in the frontal, parietal, and central regions) associated with increased workloads [32, 33, 44–46].

When lifting unknown weights, humans tend to speculate regarding their magnitude to initiate neuromuscular coordination that produces a suitable muscle recruitment strategy to control the load and maintain balance. The resulting forces are typically different from the actual forces required [6, 7, 12–14, 18, 47, 48]. Speculations regarding load magnitude, in conjunction with feedback obtained at the initiation of lifting, may be explained by the variability in the PSD of the EEG bands and the HRV.

The results of this research are limited to the specific lifting conditions in this experiment which were one lift, sagittal plan, floor-to-knuckle lifting. That notwithstanding, this research opens the door for investigating interactions related to weight knowledge under more fatiguing lifting conditions (lifting frequency, lifting duration, lifting postures, lifting heights, lifting technique, package size, and shape). Another limitation of this study is that the participants were all male college students with no experience in manual lifting tasks. Investigating a broader spectrum of the population would result in a reliable generalization of our findings.

## Conclusion

Uncertainty about the load to be lifted can be considered as a stress-adding variable in manual lifting tasks. Not knowing the weight to be lifted can trigger the muscles involved in the lifting process to respond accordingly, with greater muscle activation. This increase in the muscle activity reflects an increase in the physical demand that is required to be sustained during manual lifting.

The systemic response to lifting unknown weight results in different autonomic activities, especially with heavier weights. Lifting unknown weights also requires the utilization of more brain activity in sensorimotor areas, particularly at lower weight magnitudes, as a result of the central adaptation counteraction and speculation about the load magnitude.

Deploying EEG devices to investigate the neural responses to physical demands can provide opportunities for novel, less invasive methods of monitoring human performance. These methods are equally effective compared to traditional methods and produce reliable results.

The findings of this study stress the importance of eliminating the uncertainty associated with handling unknown loads such as in patient handling, luggage dispatching, refuse collecting, and mail distributing. This can be achieved through preliminary self-sensing of the load to be lifted, or the cautious disclosure of the actual weight of manually lifted objects, for example, through clear labeling and/or a coding system.

## Supporting information

S1 Data (ZIP)

S2 Data (ZIP)

S3 Data (ZIP)
